# Real-World Bleeding Outcomes Among Hospitalized Patients Receiving Apixaban or Rivaroxaban: A Single-Center Retrospective Cohort Study

**DOI:** 10.3390/ph19091345

**Published:** 2026-08-26

**Authors:** Cody O’Rear, Alejandro Chapa-Rodriguez, Arminder Singh, Safi Afzal, Roshan Acharya, Madalyn Wickham, Usman Younus

**Affiliations:** 1 Department of Pulmonary and Critical Care Medicine, Cape Fear Valley Medical Center, 1638 Owen Dr., Fayetteville, NC 28304, USA; achapa-rodriguez@capefearvalley.com; 2 Department of Cardiology, Cape Fear Valley Medical Center, 1638 Owen Dr., Fayetteville, NC 28304, USA; 3 Department of Internal Medicine, Bronson Health, 601 John St., Kalamazoo, MI 49007, USA; 4 Department of Pulmonary and Critical Care Medicine, Riverside Health, 500 J. Clyde Morris Blvd., Newport News, VA 23601, USA; 5 Department of Pharmacy, Atrium Health Carolinas Medical Center, 1000 Blythe Blvd., Charlotte, NC 28203, USA; 6 Department of Critical Care Medicine, Cape Fear Valley Medical Center, 1638 Owen Dr., Fayetteville, NC 28304, USA

**Keywords:** direct oral anticoagulants, apixaban, rivaroxaban, bleeding outcomes, hospitalized patients, gastrointestinal bleeding, medication safety

## Abstract

**Background**: Direct oral anticoagulants (DOACs) are widely used among hospitalized patients, yet real-world inpatient bleeding data remains relatively sparse. Most evidence derives from randomized trials enrolling ambulatory, clinically stable patients, or those being treated for a single specific indication such as acute venous thromboembolism (VTE), which may not reflect the risk profile of acutely ill inpatients with multimorbidity, procedural exposures, and fluctuating renal function. This study describes the incidence and types of bleeding events in a real-world inpatient cohort receiving apixaban or rivaroxaban at a single tertiary care center. **Methods**: We conducted a single-center, retrospective cohort study of 867 hospital encounters involving apixaban (*n* = 722, 83.3%) or rivaroxaban (*n* = 145, 16.7%) between 2019 and 2021. The primary outcome was a non-adjudicated, non-standardized composite of any documented bleeding (gastrointestinal bleeding, hematuria, intracranial hemorrhage, vaginal bleeding, or epistaxis) during the index hospitalization; International Society on Thrombosis and Haemostasis (ISTH) major bleeding or clinically relevant non-major (CRNM) bleeding definitions were not applied. Secondary outcomes included individual bleeding subtypes, hemoglobin drop ≥2 g/dL, transfusion requirement, ICU/CCU admission, vasopressor use, and bleeding-related mortality. All comparisons are unadjusted and descriptive. Between-group comparisons used Fisher’s exact test and the Mann–Whitney U test. **Results**: Bleeding was documented in 13 encounters (1.5% overall; apixaban 1.39%, rivaroxaban 2.07%); given reliance on clinical documentation rather than systematic adjudication, true incidence may be higher. The composite outcome was driven predominantly by gastrointestinal bleeding (*n* = 10, 76.9% of events). No hemoglobin drop ≥2 g/dL, transfusion, ICU/CCU admission, vasopressor use, or bleeding-related mortality occurred in either group. No statistically significant between-agent difference was observed (*p* = 0.47); all comparisons are unadjusted and descriptive. Median length of stay (LOS) differed between drug groups (8.6 vs. 6.2 days; *p* = 0.006), an exploratory finding confounded by patient class imbalance. **Conclusions**: In this single-center, retrospective cohort, bleeding was documented in 1.5% of encounters; ascertainment limitations mean true incidence may be higher. Non-adjudicated, non-standardized outcome definitions and the small number of events limit comparability with the literature and preclude definitive inference. The absence of a statistically significant between-agent difference should not be interpreted as evidence of equivalence. These findings are hypothesis-generating and should not be used for comparative inference. Prospective, multicenter studies using ISTH-standardized outcomes, indication-level data, and formal adjudication are needed.

## 1. Introduction

Anticoagulation with direct oral anticoagulants (DOACs) is increasingly common among hospitalized patients, driven by expanding indications, favorable pharmacokinetic profiles, and reduced monitoring burden compared with warfarin [1,2,3]. Despite these advantages, bleeding complications remain a clinically important concern in acutely ill, multimorbid, and elderly inpatients who face heightened risk from procedural exposures, acute organ dysfunction, and polypharmacy [2,4]. Inpatient physiology differs fundamentally from outpatient settings: acute kidney injury, variable oral intake, peri-procedural anticoagulant interruptions, and fluctuating drug exposure all influence DOAC pharmacokinetics and bleeding risk in ways that are not captured in ambulatory trial environments, making real-world inpatient safety data particularly clinically relevant [2,5,6].

Apixaban and rivaroxaban are oral direct factor Xa inhibitors that produce predictable, dose-dependent anticoagulation without routine coagulation monitoring. They differ in pharmacology in ways relevant to bleeding risk: apixaban is administered twice daily and undergoes approximately one-quarter renal elimination, whereas rivaroxaban is administered once daily for atrial fibrillation, depends more heavily on renal clearance (approximately one-third of an absorbed dose is excreted unchanged), and requires administration with food at the 15- and 20-mg doses for adequate absorption. Both are substrates of CYP3A4 and P-glycoprotein and are therefore susceptible to interactions with inhibitors or inducers of these pathways, and both require dose adjustment in renal impairment. These pharmacologic distinctions are clinically salient in hospitalized patients, in whom fluctuating renal function, variable oral intake, and polypharmacy may alter drug exposure and bleeding risk [5,6].

Although pivotal randomized trials have established favorable bleeding profiles for apixaban and rivaroxaban relative to warfarin, those trials enrolled carefully selected, clinically stable patients with atrial fibrillation or venous thromboembolism [1,3,7]. Their findings may not generalize to the heterogeneous, acutely ill populations encountered in routine inpatient care. Large real-world comparative studies, including the ARISTOPHANES analysis, the propensity-matched cohort study by Fralick et al., and registry-based cohorts such as ORBIT-AF and the Dresden NOAC Registry, have demonstrated differential bleeding profiles among DOAC agents, with several analyses suggesting lower bleeding risk with apixaban compared with rivaroxaban [8,9,10,11,12,13,14]. Real-world observational data specific to the inpatient setting remain sparse [2,15,16].

This study describes bleeding incidence among hospitalized patients receiving apixaban or rivaroxaban at a single tertiary care center. We aimed to characterize the frequency and types of bleeding events, describe patient and care-setting characteristics, and examine hospital LOS as part of a descriptive, observational safety analysis. Inpatient DOAC safety remains a clinically important but under-characterized domain, and descriptive real-world data contribute valuable context for hypothesis generation and future study design.

The objective of this study was to describe the incidence, types, and clinical characteristics of bleeding events among hospitalized patients receiving apixaban or rivaroxaban at a single center. Consistent with its retrospective, descriptive design, the study was not structured to test a prespecified hypothesis or to support comparative inference between agents.

## 2. Results

### 2.1. Cohort Characteristics

The cohort comprised 867 hospital encounters from 2019 through 2021, of which 722 (83.3%) involved apixaban and 145 (16.7%) involved rivaroxaban. The majority were Inpatient-class (814/867, 93.9%), with 49 Observation-status (5.6%), 2 Emergency (0.2%), and 2 Outpatient (0.2%) encounters. Patient class distribution differed significantly between drug groups (chi-square *p* = 0.009), with rivaroxaban encounters more frequently classified as non-inpatient. Median age was 72 years (IQR 63–80) overall and did not differ between groups (apixaban 72 (64–80); rivaroxaban 71 (61–79); *p* = 0.116). Cancer diagnoses were present in 257 encounters (29.6%) and coagulation disorders in 330 (38.1%), without significant between-group differences (*p* = 0.370 and *p* = 0.262, respectively). Baseline characteristics are presented in Table 1.

### 2.2. Bleeding Outcomes

Any bleeding occurred in 13 encounters (1.5% overall). The composite outcome was driven predominantly by gastrointestinal bleeding, which accounted for 10 of 13 events (76.9%; apixaban 8 (1.11%), rivaroxaban 2 (1.38%)). Hematuria occurred in 3 encounters (apixaban 2 (0.28%), rivaroxaban 1 (0.69%)). No intracranial hemorrhage, vaginal bleeding, or epistaxis was observed. Documented bleeding incidence was 1.39% (10/722) among apixaban encounters and 2.07% (3/145) among rivaroxaban encounters; no statistically significant difference was observed between groups (Fisher exact *p* = 0.47). This comparison is unadjusted for baseline differences and should be interpreted as descriptive only.

Among the 13 encounters with documented bleeding, median age was 73 years; a cancer diagnosis was present in 5 and a coagulation-disorder diagnosis in 5. Bleeding was predominantly gastrointestinal (10/13, 76.9%); all three hematuria events occurred in patients with a cancer diagnosis. Bleeding events occurred across the full range of observed doses, spanning prophylactic, reduced, and treatment-dose regimens.

No hemoglobin drop ≥2 g/dL, transfusion requirement, ICU/CCU admission, vasopressor use, or bleeding-related mortality occurred in either group during the index hospitalization. Bleeding outcomes are presented in Table 2.

### 2.3. Length of Stay

Median LOS was longer among apixaban encounters (8.6 days, IQR 4.1–15.3) than rivaroxaban encounters (6.2 days, IQR 3.8–11.6; *p* = 0.006). This finding is exploratory and substantially confounded by patient class: Observation-status encounters had markedly shorter LOS (median 1.7 days, IQR 1.2–2.9) than Inpatient encounters (median 8.7 days, IQR 4.8–15.4), and Observation encounters were disproportionately represented in the rivaroxaban group. LOS did not differ between encounters with and without bleeding events (*p* = 0.69). Coagulation disorder diagnoses were associated with significantly longer LOS (10.2 days, IQR 5.1–18.7 vs. 7.0 days, IQR 3.8–13.5; *p* < 0.001), reflecting underlying illness severity. LOS data are summarized in Table 3.

## 3. Discussion

In this real-world inpatient cohort, documented bleeding events were infrequent within the constraints of this dataset; true bleeding incidence may be higher than observed given reliance on clinical documentation rather than systematic adjudication. The composite outcome was driven predominantly by gastrointestinal bleeding, which accounted for nearly three-quarters of all events; isolated hematuria comprised the remainder. No intracranial hemorrhage, transfusion requirement, ICU admission, or bleeding-related mortality was observed in either drug group during the index hospitalization. Accordingly, the observed low event rate should be interpreted as reflecting documented bleeding within the EHR rather than a definitive estimate of true clinical incidence.

These descriptive findings are broadly consistent with prior observational data suggesting low short-term inpatient bleeding rates with apixaban and rivaroxaban [15,16]. Unlike pivotal randomized trials, which enrolled carefully selected, ambulatory patients with atrial fibrillation or venous thromboembolism [1,3,7], this cohort reflects routine inpatient practice including patients with acute medical conditions and multiple comorbidities. Inpatient physiology differs fundamentally from outpatient settings, with acute kidney injury, variable oral intake, peri-procedural interruptions, and fluctuating drug exposure all influencing DOAC pharmacokinetics and bleeding risk in ways that are not captured in ambulatory trial environments [5,6]. These factors underscore the clinical relevance of real-world inpatient safety data, while the methodological limitations detailed in the Limitations section constrain the strength of inference that can be drawn from this dataset.

No statistically significant difference in bleeding incidence was observed between apixaban and rivaroxaban (1.39% vs. 2.07%; *p* = 0.47). The absence of a statistically significant difference should not be interpreted as evidence of equivalence. This comparison is unadjusted; the study was substantially underpowered to detect clinically meaningful between-agent differences in a low-event-rate outcome, and exposure groups were not balanced with respect to patient class or indication. The absence of a non-DOAC comparator group further limits the ability to contextualize these bleeding rates relative to alternative anticoagulation strategies such as warfarin or low-molecular-weight heparin. Large real-world comparative studies have consistently reported differential bleeding profiles between apixaban and rivaroxaban. The ARISTOPHANES analysis, a propensity-matched study of more than 200,000 patients with non-valvular atrial fibrillation, reported significantly lower rates of major bleeding with apixaban compared with rivaroxaban [8]. Similar findings have been reported by Fralick et al. in a large propensity-matched cohort study [10], by Mamas et al. in a contemporary meta-analysis [12], and by Shurrab et al. in a population-based cohort stratified by baseline bleeding risk [13]. Registry-based studies, including ORBIT-AF and the Dresden NOAC Registry, together with more recent nationwide observational data, have likewise described differences in bleeding outcomes among DOAC agents in routine clinical practice [9,11,14,17]. The absence of a differential signal in the present study most likely reflects limited statistical power, heterogeneous and non-standardized exposure definitions, and the absence of confounder adjustment rather than true equivalence between agents.

These large-scale comparative studies are presented to provide clinical context only; the present study was not designed or powered to evaluate between-agent differences, and our findings should not be interpreted as supporting or refuting those prior observations.

In venous thromboembolism populations, randomized trials including EINSTEIN and AMPLIFY have also demonstrated favorable bleeding profiles for DOACs compared with warfarin, though agent-specific inpatient comparisons in this indication remain sparse [18,19,20]. The heterogeneity of indications in the present cohort, spanning atrial fibrillation, VTE treatment, and prophylaxis, further complicates interpretation and underscores the need for indication-stratified analyses in future research.

The absence of intracranial hemorrhage and bleeding-related mortality is clinically noteworthy, as these represent the most feared complications of anticoagulation. However, a cohort of 867 encounters with only 13 total bleeding events was substantially underpowered to detect rare catastrophic outcomes, and these null findings cannot be interpreted as evidence of safety equivalence to studies with larger event counts or longer follow-up.

The LOS difference between drug groups (8.6 vs. 6.2 days; *p* = 0.006) is best understood as an exploratory, confounded observation. Rivaroxaban encounters were disproportionately classified as Observation status, a care designation associated with markedly shorter LOS (median 1.7 days vs. 8.7 days for Inpatient encounters), and patient class was significantly imbalanced between groups. Coagulation disorder diagnoses were independently associated with longer LOS (*p* < 0.001), illustrating the role of illness severity as a key driver of hospitalization duration. LOS did not differ between encounters with and without bleeding (*p* = 0.69), suggesting bleeding was not a meaningful contributor to hospitalization length.

Indication-specific bleeding risk could not be evaluated because the cohort included patients treated for atrial fibrillation, venous thromboembolism, and thromboprophylaxis, each representing distinct clinical populations managed under different therapeutic strategies [6,20]. Dose-specific bleeding risk also could not be analyzed, which may obscure clinically meaningful differences between therapeutic and prophylactic regimens.

This analysis was restricted to bleeding-safety outcomes; thromboembolic effectiveness endpoints and anticoagulant indication were not captured and could not be evaluated. Paired safety–effectiveness assessment is an important objective for future prospective, indication-linked research.

Taken together, these findings do not identify a signal for high rates of documented bleeding within the constraints of this dataset. They should be considered hypothesis-generating rather than confirmatory; prospective research using standardized, adjudicated outcome ascertainment is needed to overcome the methodological constraints inherent to this retrospective, single-center design.

## 4. Methods

### 4.1. Study Design

This single-center, retrospective cohort study analyzed 867 consecutive hospital encounters involving apixaban or rivaroxaban therapy at a tertiary care hospital between January 2019 and December 2021. The study was designed as a descriptive, observational safety analysis; it was not intended to establish causal relationships, test comparative efficacy, or support adjusted inference between anticoagulant agents. The unit of analysis was the hospital encounter; patients contributing multiple encounters were counted separately for each admission.

The dataset did not include a patient-level identifier, so encounters could not be linked across admissions; the number of unique patients and repeated hospitalizations could not be determined. Repeated encounters from the same patient may introduce clustering that was not accounted for.

### 4.2. Study Population

Adult patients (≥18 years) who received at least one documented dose of apixaban or rivaroxaban during a hospital encounter were eligible. Patients were identified using electronic health record (EHR) medication administration data. Encounters were classified by patient class (Inpatient, Observation, Emergency, or Outpatient) as recorded in the EHR. The final analytic cohort comprised 867 hospital encounters.

### 4.3. Exposure Definition

The primary exposure was the DOAC agent, categorized as apixaban or rivaroxaban based on the medication administered. Exposure was defined as receipt of at least one inpatient dose and does not account for duration of therapy, timing relative to any bleeding event, or prior outpatient DOAC use. The range of doses observed, including apixaban 10 mg (VTE treatment loading dose, *n* = 99) and rivaroxaban 2.5 mg (ACS/CAD vascular dose, *n* = 6), indicates a heterogeneous mix of indications. Anticoagulant indication was not captured in the dataset.

### 4.4. Outcome Definitions

#### 4.4.1. Primary Outcome

Any bleeding (composite), defined as documentation of at least one of the following during the index hospitalization: gastrointestinal bleeding, hematuria, intracranial hemorrhage, vaginal bleeding, or epistaxis. Bleeding events were not adjudicated using standardized criteria such as ISTH major bleeding or clinically relevant non-major bleeding (CRNM) definitions [21]. This limits direct comparability with prior studies that applied formal adjudication frameworks.

#### 4.4.2. Secondary Outcomes

Individual bleeding subtypes; hemoglobin drop ≥2 g/dL from baseline; transfusion requirement; ICU/CCU admission; vasopressor requirement; bleeding-related mortality; and hospital LOS.

### 4.5. Data Abstraction

Binary outcome variables were abstracted from structured EHR fields and free-text clinical documentation. Variables were coded as present (1) when documented and absent (0) when not documented, reflecting a standardized abstraction protocol applied uniformly across all encounters to minimize differential misclassification between exposure groups. Coagulation disorder was defined based on EHR-coded diagnoses (e.g., thrombophilia, coagulopathy, or prior bleeding disorder), though granularity was limited to the ICD-coded level and specific subtypes were not further characterized. Because the coding protocol records only documented events, reliance on clinical documentation rather than systematic adjudication may result in under-ascertainment, particularly for hemoglobin trends and transfusion requirements. Antiplatelet agent use was a planned covariate but was not reliably captured in the source data and could not be analyzed. LOS was computed from admission and discharge timestamps.

### 4.6. Statistical Analysis

Continuous variables are reported as median with interquartile range (IQR). Categorical variables are reported as counts and percentages. Between-group comparisons used Fisher’s exact test (categorical) and the Mann–Whitney U test (continuous). Patient class distribution was compared using the chi-square test. All analyses are unadjusted and descriptive; *p*-values are reported for transparency but do not imply inferential significance and should be interpreted cautiously in an underpowered dataset where no causal inference is intended. Multivariable regression was not performed due to the limited number of bleeding events (*n* = 13), which precluded meaningful adjustment. Bleeding events were not time-stamped relative to anticoagulant initiation, precluding time-to-event analysis. All tests were two-sided with a significance threshold of *p* < 0.05.

Because the cohort comprised all consecutively eligible encounters, no a priori sample-size calculation was performed. To characterize the precision of the between-agent comparison, we estimated detectable effect sizes by Monte Carlo simulation (20,000 iterations) of Fisher’s exact test under the observed group sizes (722 apixaban, 145 rivaroxaban) and the observed apixaban bleeding rate (1.39%). At a two-sided α of 0.05, the study had approximately 80% power only to detect a rivaroxaban bleeding incidence of roughly 6% or greater (relative risk ≈ 4 versus apixaban). Analyses and simulations were performed in Python LOS 3.12 (SciPy 1.11).

### 4.7. Manuscript Preparation

During the preparation of this manuscript, ChatGPT version 4o (GPT-4o) was utilized for the purposes of generating the reference list and in-text citations for this manuscript. This was accomplished by uploading a list of the referenced sources as well as a copy of the manuscript. A text prompt was then provided to generate a list of references from the provided sources, in NLM format, as well as to insert appropriate in-text citations into the manuscript based on those sources. All of the AI-generated output was reviewed at length and edited where appropriate to ensure the content of the manuscript is factual and accurate.

## 5. Limitations

This study has several important limitations. First, the primary outcome was defined using a broad, non-adjudicated composite that does not align with established bleeding classification frameworks such as ISTH major bleeding or CRNM criteria. The use of non-standardized outcome definitions limits direct comparability with the published literature and may dilute event severity by combining clinically consequential events with minor bleeding manifestations. Second, outcome identification relied entirely on clinical documentation rather than systematic adjudication; the absence of hemoglobin decline, transfusion, and critical bleeding events raises the possibility of under-ascertainment resulting from documentation gaps rather than true clinical absence. Third, selection bias is likely present: patients receiving DOAC therapy during hospitalization may represent a clinically more stable subset relative to those managed with alternative anticoagulation strategies, which would tend to favor lower observed bleeding rates.

Fourth, all between-group comparisons are unadjusted. Despite appropriate restraint from multivariable modeling given the low event count, the descriptive comparisons between apixaban and rivaroxaban remain vulnerable to confounding by indication, illness severity, and care setting. Fifth, exposure classification based on receipt of any inpatient dose does not account for duration of therapy, timing relative to any bleeding event, or prior outpatient DOAC use, and may misclassify the nature and extent of anticoagulation exposure. Sixth, the inability to account for renal function, concomitant antiplatelet therapy, and baseline anemia, which are key determinants of DOAC-associated bleeding risk, substantially limits bleeding risk interpretation.

Seventh, anticoagulant indication was not captured, and dose heterogeneity indicates a mix of indications (atrial fibrillation, VTE treatment, and prophylaxis) with materially different bleeding risk profiles that could not be formally analyzed. Dose-specific bleeding risk could not be assessed, obscuring potential differences between treatment and prophylactic regimens. Eighth, the dataset comprises hospital encounters rather than unique patients; repeated encounters from the same patient may introduce clustering effects that were not accounted for and could bias variance estimates. Ninth, the absence of temporal linkage between anticoagulant administration and bleeding events precludes causal inference and makes time-to-event analysis inappropriate. Tenth, follow-up was limited to the index hospitalization; post-discharge bleeding events were not captured, which may underestimate the true burden of bleeding in patients continuing DOAC therapy after discharge. Finally, LOS analyses are exploratory and substantially confounded by patient class imbalance and illness severity, limiting their interpretability as pharmacologic observations.

## 6. Conclusions

In this single-center, retrospective cohort of 867 hospital encounters, bleeding was documented in 1.5% of cases; ascertainment limitations mean true incidence may be higher. No hemoglobin drop ≥2 g/dL, transfusion requirement, ICU/CCU admission, vasopressor use, or bleeding-related mortality occurred during the index hospitalization. The composite outcome was driven predominantly by gastrointestinal bleeding. No statistically significant difference in bleeding incidence was observed between apixaban and rivaroxaban, and the absence of a significant difference should not be interpreted as evidence of equivalence. All comparisons are unadjusted and descriptive, and the study was underpowered to detect clinically meaningful between-agent differences. Non-standardized outcome definitions and possible under-ascertainment further limit the strength of inference. These findings are hypothesis-generating. Prospective, multicenter studies using ISTH-standardized outcome criteria, indication-level data, formal adjudication, and longer follow-up are needed to more definitively characterize real-world inpatient DOAC safety and support meaningful between-agent comparisons.

## Figures and Tables

**Table 1:** Baseline Characteristics by Anticoagulant Agent.

Characteristic	Apixaban (*n* = 722)	Rivaroxaban (*n* = 145)	*p* Value
Age, median (IQR), years	72 (64–80)	71 (61–79)	0.116
**Patient class, *n* (%)**			
Inpatient	682 (94.5%)	132 (91.0%)	0.009 ^a^
Observation	38 (5.3%)	11 (7.6%)	
Emergency/Outpatient	2 (0.3%)	2 (1.4%)	
Cancer diagnosis, *n* (%)	219 (30.3%)	38 (26.2%)	0.370
Coagulation disorder, *n* (%)	281 (38.9%)	49 (33.8%)	0.262
Median LOS, days (IQR)	8.6 (4.1–15.3)	6.2 (3.8–11.6)	0.006

^a^ Chi-square test. All other *p* values from Fisher’s exact test (categorical) or Mann–Whitney U test (continuous). IQR, interquartile range; LOS, length of stay.

**Table 2:** Bleeding Outcomes by Anticoagulant Agent.

Outcome	Apixaban (*n* = 722)	Rivaroxaban (*n* = 145)	*p* Value
**Any bleeding** (**composite**)**, *n*** (**%**)	10 (1.39%)	3 (2.07%)	0.47
GI bleeding, *n* (%)	8 (1.11%)	2 (1.38%)	1.00 ^b^
Hematuria, *n* (%)	2 (0.28%)	1 (0.69%)	0.40 ^b^
Intracranial hemorrhage, *n* (%)	0 (0.00%)	0 (0.00%)	N/A
Vaginal bleeding, *n* (%)	0 (0.00%)	0 (0.00%)	N/A
Epistaxis, *n* (%)	0 (0.00%)	0 (0.00%)	N/A
Hgb drop ≥ 2 g/dL, *n* (%)	0 (0.00%)	0 (0.00%)	N/A
Required transfusion, *n* (%)	0 (0.00%)	0 (0.00%)	N/A
ICU/CCU admission, *n* (%)	0 (0.00%)	0 (0.00%)	N/A
Required vasopressors, *n* (%)	0 (0.00%)	0 (0.00%)	N/A
Bleeding-related mortality, *n* (%)	0 (0.00%)	0 (0.00%)	N/A

^b^ Fisher’s exact test. N/A, not applicable (zero events in both groups). GI, gastrointestinal; ICU, intensive care unit; CCU, cardiac care unit; Hgb, hemoglobin.

**Table 3:** Length of Stay by Group (Exploratory).

Group	Median LOS, Days (IQR)	*p* Value
Apixaban (*n* = 722)	8.6 (4.1–15.3)	0.006 vs. rivaroxaban
Rivaroxaban (*n* = 145)	6.2 (3.8–11.6)	
Bleeding events (*n* = 13)	8.1 (4.4–18.1)	0.69 vs. no bleeding
No bleeding event (*n* = 854)	8.1 (4.1–15.2)	
Coagulation disorder (*n* = 330)	10.2 (5.1–18.7)	<0.001 vs. no disorder
No coagulation disorder (*n* = 537)	7.0 (3.8–13.5)	

All *p* values from Mann–Whitney U test. LOS analyses are exploratory and unadjusted. IQR, interquartile range.

## Data Availability

The data presented in this study are available on reasonable request from the corresponding author. The data are not publicly available because they were derived from patient electronic health records and are subject to patient privacy, confidentiality, and institutional restrictions.
